# Child injuries in Lebanon: assessing mothers’ injury prevention knowledge attitude and practices

**DOI:** 10.1186/s40621-023-00434-9

**Published:** 2023-06-20

**Authors:** Samar Al-Hajj, Rawan El Haj, Monique Chaaya, Rana Sharara-Chami, Amber Mehmood

**Affiliations:** 1 Epidemiology and Population Health Department, Faculty of Health Sciences, American University of Beirut, PO Box 11-0236, Riad El-Solh, Beirut, 1107 2020 Lebanon; 2 Faculty of Medicine, American University of Beirut, Beirut, Lebanon; 3 College of Public Health, University of South Florida, Tampa, FL USA

**Keywords:** Child injury, Injury prevention, Injury control, Lebanon, KAP study

## Abstract

**Background:**

Childhood injury is a neglected public health problem with a sizeable burden on children’s well-being and their families. This study aims to describe the pattern and types of childhood injuries and to determine the level of mothers’ Knowledge, Attitude, and Practices (KAP) towards childhood injury prevention in Lebanon. The study further examines the association between childhood injury occurrence and mothers’ supervision.

**Methods:**

This cross-sectional study recruited mothers of children aged up to 10 years from multiple sites (i.e., a medical center, a private clinic, a healthcare facility, and a refugee camp clinic). Data were collected on mothers’ KAP toward childhood injuries using self-administrated questionnaires. A summation score for KAP correct answers was calculated and descriptive and statistical analyses were performed to measure the association between the outcomes.

**Results:**

A total of 264 mothers were surveyed and injury data were collected on their 464 children. The prevalence of childhood injury was 20% in the past 12 months, mostly sustained by males (53.8%) and children aged 5–10 years (38.7%). The most common type of injury was fall (48.4%), followed by burns (%7.5), and sports injuries (7.5%). Hospitalized children were more likely to be males and older than 5 years (*p* < 0.001). More than one-third of the mothers demonstrated poor knowledge, while the majority showed poor practice (54.4%), and fair attitude (45.6%) towards child injury prevention. Children of working mothers have three times higher odds of sustaining injuries (OR: 2.95, 95% CI: 1.60;5.47) compared to those of non-working mothers, accounting for possible confounders (*p* = 0.001).

**Conclusion:**

Childhood injuries represent a major health problem in Lebanon. Findings from this study showed that mothers are less knowledgeable and unprepared to prevent their children from getting injured. Educational programs are much needed to address the gap in the mothers' KAP toward child injury prevention. Further studies are recommended to understand the cultural context and examine its key determinants to identify effective strategies and develop tailored interventions for preventing childhood injuries.

## Background

Unintentional injury is one of the leading causes of mortality and morbidity among children, accounting for almost 90% of the global child death rate (Forjuoh 2012; Prinz 2008). In 2016, over 600,000 children aged 14 years and below died as a result of unintentional injuries worldwide (World Health Organization 2018). In addition to its large impact on a child’s health and long-term mental and functional impairment, childhood injuries impose a substantial economic burden on families and drain essential resources from healthcare systems, especially in limited-resource settings (Rivara et al. 1989; CfDCP 2012). Unintentional injuries such as falls, road traffic injuries, burns, drowning, and poisoning, are common during childhood, particularly for children aged 10 years and younger. Due to their curious and exploratory nature, children are more prone to injuries. Children’s tendency to engage in risky behaviors and underestimation of their surrounding hazards, place them at an increased risk of sustaining unintentional injuries. These injuries vary considerably in severity and outcomes, often requiring medical attention through emergency department visits and lengthy hospitalization, and resulting in short- or long-term disabilities (Sariaslan et al. 2016).

The burden of childhood injury is disproportionally distributed between developing and developed countries with the majority of child injury-related mortality and morbidity occurring in low- and middle-income countries (Orton et al. 2014). Middle Eastern studies had underscored the gravity and the increased burden of childhood injuries in the Eastern Mediterranean region (Adeloye et al. 2018; Halawa et al. 2015; Mehmood et al. 2018; Al-Hajj et al. 2020a). Compared to its neighboring countries, Lebanon sustains a larger burden of childhood injuries, with an estimated 11% injury-related death of the total number of deaths for children under 5 years, compared to a 3% average in Eastern Mediterranean countries (Samad 2008). Childhood injury claims a large proportion of the child death burden in Lebanon, estimated to be the 3rd leading cause of death and the 6th leading cause of disability-adjusted life years (DALYs) among children under the age of 14 years (Kassebaum et al. 2017). Despite the large toll of child injury and the high proportion of children in the Lebanese population, studies investigating childhood injuries remain scarce and insufficient in scope and policy impacts (Nuwahid et al. 2002; Al-Hajj et al. 2020b).

An emerging body of literature has identified multiple factors associated with childhood injuries: child’s age, gender, race, family socioeconomic status, adult supervision, lack of parental injury awareness, absence of home safety proofing behavior, and limited access to healthcare services (Orton et al. 2014; Halawa et al. 2015; Qasem et al. 2017). Lack of caregiver supervision has been implicated as a contributory factor in many childhood injuries such as falls, road traffic injuries, drowning, choking, and playground injuries (Barton and Schwebel 2007). Some studies indicated the association of home injuries with the mothers’ working status (Morrongiello and Schell 2010; Saluja et al. 2004; Munro et al. 2006), particularly as many injuries are attributed to the lack of attention and continuous supervision by the substitute caregivers. Similar studies further identified a strong correlation between childhood injuries and a mother’s professional working status as a proxy for the lack of direct parental supervision at home (McMunn et al. 2012; Karadeniz and Kahriman 2018).

A limited number of studies have investigated parental knowledge and attitudes toward childhood injury and safety practices in the Eastern Mediterranean region (Samad 2008; Nuwahid et al. 2002). To the best of our knowledge, there is a dearth of studies that explored mothers’ knowledge, attitude, and practices (KAP) of childhood safety and injury prevention. Moreover, among the Eastern Mediterranean countries, Lebanon is one of the countries with the highest proportion of working women; around 31.77% in 2020, making it a vital factor that impacts caregivers’ supervision practices and possibly increases the prevalence of child injuries, particularly as working mothers have a better understanding of injury prevention concepts. Therefore, there is an urgent need to define and adopt childhood injury prevention strategies in this sociocultural context (Annahar. 2017).

This study aims to describe the magnitude and pattern of childhood injury in Lebanon and to examine mothers’ level of knowledge, attitude, and practice of childhood safety and injury prevention. The study further aims to measure the association between the occurrence of child injuries and the mothers’ working status. The main hypothesis of the study assumed that children of working mothers are more likely to sustain an injury compared to those of non-working mothers, accounting for possible confounders. The outcomes of this study will help to gain insights into the key determinants for child injuries and identify effective preventive strategies.

## Methods

### Study design

A cross-sectional study was conducted between January and March 2020 using a self-administrated survey, directed to mothers older than 18 years and who have at least one child aged 10 years or below. This study adopted the knowledge, attitude, and practices (KAP) framework to assess mothers’ KAP in preventing such injuries. The original KAP framework was introduced by Schwartz (1976) to capture KAP of Canadian public health nurses, where 4 domains were presented: demographic characteristics, knowledge statements, Attitude statements, and practice statements. This framework presents the human behavior change process, classified into three domains: acquiring knowledge, generating attitudes/beliefs, and forming practice/behaviors, thus playing a remarkable role in preventing public health issues. Since childhood injuries are preventable health problems, we adapted the KAP model to the Eastern Mediterranean context and used it to understand mothers’ KAP toward child injury prevention. The American University of Beirut Institutional Review Board (IRB) approved the study (SBS-2019-0145).

### Participants

The study was directed at working and non-working mothers, from diverse backgrounds and socioeconomic statuses, attending healthcare facilities and educational institutions across multiple sites in the capital city Beirut, Lebanon.

### Sampling strategy

To accommodate multiple perspectives, we adopted a maximum variation sampling strategy for recruitment. We surveyed mothers from multiple locations in the greater Beirut city including a medical center, an educational institution, a private clinic, a healthcare facility, and a refugee camp, and a United Nations Relief and Works Agency for Palestine Refugees (UNRWA) clinic (patients or accompanying relatives). The sample size was calculated based on an effect size equal to 0.2, with a 95% level of confidence, a 5% margin of error, a 70% response rate, and a 50% prevalence of injury, using the formula: Sample size = [(1.96)^2^* prevalence* (1)]/ (margin of error)^2^. Considering the power of the study, the statistically significant result in the primary outcome (occurrence of childhood injuries) would demonstrate practicality in finding a difference between groups as the sample size needed was increased.

### Participant recruitment

At the waiting rooms of the selected sites, data collectors approached mothers who were presented to any of the selected healthcare facilities with or without any medical complaint related to injuries sustained by the mothers, their children, or any other family member. Mothers were randomly selected (every other woman to avoid selection bias) and directly asked to participate in the study if they are interested and they match the selection criteria. The study team explained the study objective and solicited the eligible candidates’ oral consent to participate in a (self-administered paper-based/online) survey, which was administered at the same time and location/ sent via email/ or mailed to mothers. At the educational institution, female workers were randomly selected (every fifth working mother on the list) from the institution’s human resources mailing list. The survey package was emailed to mothers; it included a descriptive summary page of the study and a consent form.

### Survey tool

The used survey tool was adopted from previous studies, adjusted to reflect the Lebanese context, and translated into Arabic (Nour et al. 2018; Guilfoyle 2009; Morrongiello and Corbett 2006). The survey encompasses 3 main sections with a corresponding sub-set of 69 questions to capture: (1) Mother’s socio-demographic characteristics (i.e. educational level, work status, marital status, number of children, and monthly family income classified as low (< 675,000 LBP: < 338 USD), middle (675,000–2,000,000 LBP: 338 UD-1,000USD), or high; (> 2,000,000 LBP: > 1,000 USD),) Rates are based on March 2020 conversion rate), (2) Child’s injury history in the last 12 months (i.e., injury type, mechanism, location, and severity classified as mild, moderate and severe), and (3) Mother’s KAP towards injury prevention (i.e. beliefs, attitudes, perceptions, and knowledge of childhood injury prevention, adopted safety measures, and access to child safety resources). Three subsets of questions investigated the mothers’ KAP towards child injury prevention: their engagement in specific practices such as leaving their child in the water tub without adult supervision or leaving the electric outlets without any covers or switch plates, their attitude towards particular behaviors that reduce the severity of child injuries such as whether a first aid kit is needed to be in the house or not, and their knowledge about the best practices to prevent child injuries such as where to store poisonous and chemical materials. The survey was pilot tested to ensure face validity and applicability in the Lebanese context. The Cronbach’s alpha coefficient was 0.67, knowing that the tool was already tested for construct validity in a different study in the Arab region (Saudi Arabia) (Nour et al. 2018). The questions were rated on the 5-item Likert scale (for practice section: never, rarely, sometimes, most of the time, always/for attitude section: strongly disagree, disagree, neither agree nor disagree, agree, strongly agree), then ‘Poor’ score was given less than 50% correct/positive answers, ‘Fair’ for 50–75% correct/positive answers, and ‘Good’ for more than 75% correct/positive answers. This scoring summation was adopted and validated by similar studies (Nour et al. 2018; Eldosoky 2012; Siddiqui et al. 2018).

### Data analysis

The overall response rate was 60%. The data was cleaned, tabulated, and analyzed using STATA (Version 14). We performed descriptive statistics to describe the mothers' socio-demographic characteristics and to identify the children’s injury mechanisms, frequencies, and percentages. Concerning the mothers' KAP toward child injury prevention, we calculated the score as the summation of correct/positive answers. We further conducted a Chi-square test to measure the association between the occurrence of child injuries and the mothers’ working status (McHugh 2013). We used simple logistic regression to determine the unadjusted odds ratio (OR) and 95% confidence interval (CI), and to obtain the p-values, accounting for the clustering effect (household). The significance level was determined at p-value < 0.05. We performed multiple logistic regression analyses to obtain the adjusted ORs. To detect possible confounders, the adjusted and unadjusted ORs were compared. The best model that tested this project's hypothesis was selected based on its significance (*p*-value < 0.05) and its goodness of fit (*p*-value > 0.05) (Archer and Lemeshow 2006) (Please refer to “Appendix” section).

## Results

This study included 264 mothers, reporting on 464 children aged up to 10 years.

### Mothers characteristics

As reported in Table 1, interviewed mothers had a mean age of 32.7 ± 6.6 years with a majority in the age group of 35 years and above (40.5%, N = 107), lived with a spouse (92.6%), and had one or two children aged 10 and below (40.4% and 41.6%, respectively). More than half of the mothers (56.9%) are university graduates. Nearly 46.4% reported high monthly family income, 23.6% reported middle income, while 30% reported low income.

**Table 1:** Characteristics of mothers with children 10 years and younger (N = 264, year: 2020)

Variable	N	%
Mothers' age (years)		
< 25	25	9.5
25–< 30	65	24.6
30–< 35	67	25.4
≥ 35	107	40.5
Mother's highest educational level		
Below secondary level	70	26.9
Secondary level	42	16.2
University and above level	148	56.9
Mothers' marital status		
No spouse	19	7.3
With spouse	241	92.7
Housing status		
Rented	104	39.9
Owned	146	55.9
Others	11	4.2
Income level		
Low income	66	30.0
Middle income	52	23.6
High income	102	46.4
Number of children 10 years and below		
One	103	40.4
Two	106	41.6
Three	36	14.1
Four or more	10	3.9
Mothers' working status and working details		
Ever worked mother		
No	113	43.8
Yes	145	56.2
Mothers' current working status		
No	134	51.9
Yes	124	48.1
Mothers’ work domain *		
Health, science, and pharmaceuticals	40	47.6
Business and administration	16	19.1
Education	10	11.9
Others	12	14.3
Accounting and banking	4	4.8
Engineering and manufacturing	1	1.2
Human resources	1	1.2
Working hours/day*		
2–4 h	11	8.9
5–8 h	52	42.3
More than 8 h	60	48.8
Mothers' KAP towards child injury prevention		
Mothers' knowledge		
Poor knowledge	94	35.8
Fair knowledge	151	57.4
Good knowledge	18	6.8
Mothers' attitude		
Poor attitude	38	14.5
Fair attitude	120	45.6
Good attitude	105	39.9
Mothers' practice		
Poor practice	143	54.4
Fair practice	99	37.6
Good practice	21	8.0

*Totals do not add up to 100% (264) due to missing value

Income and its equivalence in USD: Low (< 675,000 LBP: < 338 USD), Middle (675,000–2,000,000 LBP: 338 UD-1,000USD), High (> 2,000,000 LBP: > 1,000 USD), rates are based on March 2020 conversion rate, prior to currency inflation

poor: < 50% of the items were correctly/positively answered, Fair: 51–75% of the items were correctly/positively answered, Good: > 75% of the items were correctly/positively answered

Approximately 48.1% were working mothers with 40% of them working in the health, science, and pharmaceutical fields (40%). 48.8% were working more than 8 h per day, and 81.2% were in the private sector with a mean of 12.8 ± 7.8 years of work experience.

### Mothers’ knowledge, attitude, and practices towards child injury prevention

In general, participating mothers showed fair knowledge (57.4%) with a mean of 8.5 (± 2.7) out of 14, fair attitude (45.6%) with a mean of 5 (± 1.9) out of 10, and poor practice (54.4%) with a mean of 13.1 (± 4.6) out of 25 toward child injury prevention. More than one-third of the mothers reported poor knowledge (35.7%) (Table 1).

Most mothers knew the appropriate actions to be done when the child is injured by an electrical short circuit (74%), in a gas leak (72%), or has mild or moderate burns (66%). While 71% recognized the ideal place to store cleaning products and detergents, only 61% knew the immediate response in case of chemical poisoning. Only 65% of the mothers were knowledgeable about the child’s car safety laws. Specifically, 42% correctly stated the minimum age children become eligible to use a seatbelt, while only 13% knew the age a baby should stay riding in a rear-facing child safety seat. Despite reasonable literacy levels, only 12% of the mothers accurately identified the water level that causes drowning of children aged less than 4 years (Table 2).

**Table 2:** The percentage of mothers reporting correct/positive KAP answers on child injury prevention

Items testing mothers' KAP towards child injury prevention	% of mothers reporting correct answers
*Items testing mothers' knowledge towards child injury prevention*	
Mother knows what to do when a child is injured by an electrical short circuit	74
Mother knows what to do in the event of a gas leak	72
Mother knows what the best place is to store cleaning products and detergents	71
Mother knows what to do if a child has a case of mild or moderate burn	66
Mother knows whether there is a child car safety law in Lebanon	65
Mother knows what to do in the event of chemical poisoning	61
Mother knows the speed limit on the highways in Lebanon	47
Mother knows the minimum age when children are ready to be seated with only a seat belt	42
Mother knows the age to which a baby should ride in a rear-facing child safety seat	13
Mother knows that children less than 4 years can drown in as little as a small amount of water	12
*Items testing mothers' attitude/attitude towards child injury prevention*	
Mother is aware of the importance of training others in the home on first-aid	96
Mother is aware of the importance of owing first aid kit in every home	94
Mother is aware of the importance of getting information about how to deal with child injuries	93
Mother is aware of the necessity to take all preventive measures to prevent child home injuries	91
Mother believes that the taken actions as a parent can protect the child from accidents	81
Mother is willing to undergo home safety training	78
Mother believes that domestic injuries affect your child psychologically	75
Mother trusts her ability to act appropriately in emergencies	67
Mother believes that the surrounding affects the way she keeps her child safe	42
Mother believes that her surrounding will blame her for her child’s injuries	33
Mother believes that children’s accidents are manageable and easily solved	32
Mother believes that it is her fault if her child gets injured	26
Mother believes that fortune and fate play a big part in determining whether or not the child gets injured	25
*Items testing mothers' practice towards child injury prevention*	
Not leaving a child under 10 at home alone	88
Checking gas leak from a gas cylinder	88
Taking preventive measures to protect own child	83
Not leaving the child alone in a tub of water	82
Locking cabinets with medicines and cleaners or store them out of reach	82
Adults taking care of the child do not leave them alone	82
Children do not have access to standing water with no adult supervision	80
Not leaving the child alone on the bed	40
Having first aid training at home	35
Keeping electrical outlets plugged with covers	33
Not connecting multiple devices with one socket	21
Having a fire extinguisher at home	18
*Mothers' practices among families that own cars*	
Using a seatbelt	84
Not leaving the child alone in the car	78
Not placing the child on laps while in the car	62
Asking others to put away their phone while driving	53
Using a car booster seat for your child	37
*Mothers' practices (applicable to mothers who drive)*	
Not drinking while driving	97
Not breaking traffic regulations	88
Not speeding while driving with a child on board	77
Not texting while driving	64
Not calling while driving	45

Considering the social and cultural structure of the Lebanese households, 42% of the mothers reported that they were influenced by social norms when it comes to keeping their children safe. Furthermore, 75% were aware of the psychological distress their children suffer from due to injuries. Only 26% of the mothers blamed themselves for their children’s injuries, while 25% believed that fate vastly contributes to the child’s injuries. (Table 2).

Most participating mothers demonstrated good practices toward child injury prevention, including frequently checking for gas leaks (88%), constantly supervising their children in a water tub (82%), and locking medicines and toxic products. Yet only 18% owned a fire extinguisher at home. Nonetheless, only 40% of the mothers refrain from leaving their children alone in bed, 35% owned a first aid kit at home, 33% kept electrical outlets plugged with covers, and 21% avoided connecting multiple devices with one socket (Table 2).

Eighty-four percent of mothers showed good practices for using a seatbelt and, 78% confirmed not leaving their children alone in the car. However, only 37% used the child restraint system. While 97% of the driving mothers did not drink alcohol, 88% admitted to violating the traffic laws and 77% confessed to exceeding the speeding limit. More than 55% of the mothers reported using their cell phones while driving, even when their children are on board, which could potentially be an area of targeted behavioral intervention (Table 2).

### Characteristics of children’s injury patterns

Nearly one-fourth of children (19.3%) experienced injuries in the past 12 months, 20.7% of whom sustained severe injuries (Table 3). More than half of the children (53.5%) were males, with a mean age for injured children of 5.2 years (± 2.9). Children were distributed across age groups with 3–5 (30%), 7–10 (28.3%), 0–3 (22.1%), and 5–7 (19.1%) (Table 3).

**Table 3:** The distribution of children aged ≤ 10 years by age, gender, occurrence, and severity of injury in the past 12 months (N = 464; year:2020)

Variable	N	%
*Child age categories*		
< 3 years	102	22.1
3- 5 years	141	30.5
> 5–7 years	88	19.1
> 7 years	131	28.3
*Child gender*		
Female	214	46.5
Male	246	53.5
*Child injury in the past 12 months*		
No injury	371	80.7
Injuries	89	19.3
*Severe injury in the past 12 months among injured children (applicable n* = *89)*		
No	69	79.3
Yes	18	20.7

*Not all variables add to the total of 464 due to missing values

**Fig. 1:**
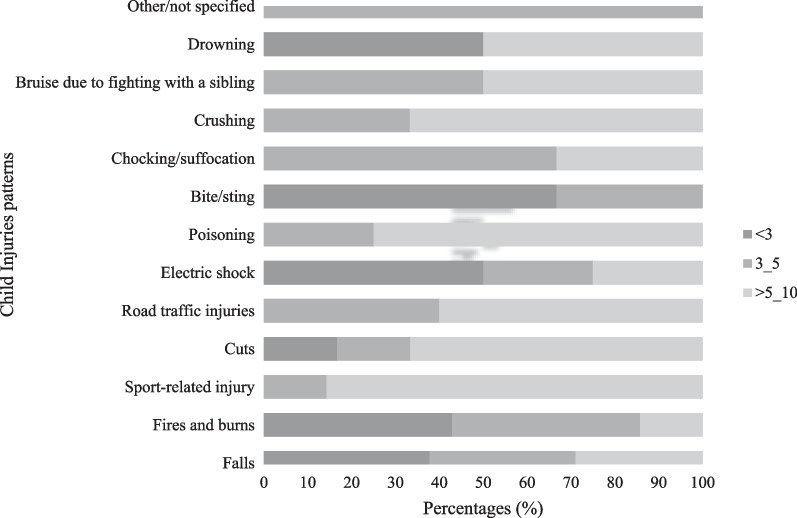
Injuries patterns by children's age groups (years)

Fall injury was reported as the leading cause of childhood injury (48.4%), followed by burns (7.5%) and sports-related injuries (7.5%) (Table 4). Drowning caused 2.2% of the injuries. The majority of the fall injuries were sustained by children less than 3 years old, while older children (> 5 years) mainly experienced road-traffic and sports-related injuries (Fig. 1).

**Table 4:** The distribution of reported child Injuries based on child gender and age (N = 93 injuries) *

Child injury	Falls N (%)	Burns N (%)	Sport- injury N (%)	Road injuries N (%)	Poisoning N (%)	Drowning N (%)	Others combined** N (%)	Total N (%)
	45 (48.4)	7 (7.5)	7 (7.5)	5 (5.3)	4 (4.3)	2 (2.2)	23 (24.7)	93 (100)
*Gender*								
Females N (%)	23 (51.1)	4 (57.1)	0 (0)	2 (40)	3 (75)	1 (50)	10 (43.5)	43 (46.2)
Males N (%)	22 (48.9)	3 (42.9)	7 (100)	3 (60)	1 (25)	1 (50)	13 (56.5)	50 (53.8)
*Age*								
0–3	17 (37.8)	3 (42.9)	0 (0)	0 (0)	0 (0)	1 (50)	5 (21.8)	25 (26.9)
3–5	15 (33.3)	3 (42.9)	1 (14.3)	2 (40)	1 (25)	0 (0)	9 (39.1)	32 (34.4)
5–10	13 (28.9)	1 (14.2)	6 (85.7)	3 (60)	3 (75)	1 (50)	9 (39.1)	36 (38.7)

*Five children reported two types of injuries

**Others Combined: electric shock, bite/sting, chocking, crushing, not specified

It is worth noting that five children have experienced multiple injuries (i.e., two types of injuries). Electric shocks and sports-related injuries were only reported in males (100%), while burns and poisoning were mostly sustained by females with a high prevalence of 57.1% and 75%, respectively (Table 4).

### Effect of mothers’ working status, educational level, and family income on child injury risk

Adjusting for the clustering effect, only the mother’s working status, educational level, and family income revealed a statistically significant association with child injury in the bivariate analyses (*p*-value < 0.05) (Table 5). Children of currently working mothers were 2.6 times more likely to sustain an injury compared to those of non-working mothers (*p*-value < 0.05). The odds of sustaining a child injury among mothers with middle- and high-income were almost 4 and 5.4 times, respectively, compared to that of children in low-income families. Moreover, children, whose mothers have a university diploma, were three times more likely to get injured compared to those whose mothers did not attend universities.

**Table 5:** Bivariate analyses between the occurrence of child injuries, mothers' working status, and selected covariates adjusted for clustering (Household) effect

Variable	Unadjusted OR	95% CI of OR	P-value
*Current working mothers (Yes/No)*			
No	1		
Yes	2.61	(1.51;4.52)	0.001*
*Ever worked status*			
No	1		
Yes	2.72	(1.55;4.74)	0.000*
*Mothers' age*			
< 25	1		
25–< 30	0.68	(0.23;1.99)	0.483
30– < 35	1.33	(0.47;3.80)	0.590
≥ 35	1.36	(0.51;3.66)	0.540
Mothers profession	1.15	(0.91;1.45)	0.227
*Working hours/day*			
2–4 h	1		
5–8 h	1.79	(0.43;7.39)	0.424
More than 8 h	1.80	(0.43;7.47)	0.418
*Working sector*			
Non-Private sector	1		
Private sector	1.13	(0.40;3.12)	0.821
*Mother's educational level*			
Below secondary level	1		
secondary level	1.98	(0.86;4.58)	0.106
University level	2.97	(1.49;5.19)	0.002*
*Mothers' marital status*			
Without spouse	1		
With spouse	0.64	(0.22;1.86)	0.412
*Housing status*			
Rented	1		
Owned	1.47	(0.83;2.59)	0.185
Other	0.90	(0.27;2.95)	0.860
*Family income*			
Low income	1		
Middle income	4.11	(1.65;10.2)	0.002*
High income	5.37	(2.27;12.9)	0.000*
*Number of children 10 years and below*			
One	1		
Two	1.40	(0.75;2.60)	0.291
Three	0.69	(0.29;1.66)	0.410
Four or more	0.35	(0.11;1.09)	0.071
*Child gender*			
Female	1		
Male	0.97	(0.59;1.58)	0.909
*Child age*			
< 3 years	1		
3–5 years	1.33	(0.71;2.51)	0.376
> 5–7 years	0.83	(0.38;1.76)	0.615
> 7 years	0.95	(0.48;1.86)	0.468
*Mothers' practice*			
Poor practice	1		
Fair practice	1.19	(0.68;2.11)	0.676
Good practice	0.23	(0.07;1.49)	0.149
*Mothers' attitude*			
Poor attitude	1		
Fair attitude	1.30	(0.61;2.79)	0.495
Good attitude	1.21	(0.54;2.71)	0.637
*Mothers' knowledge*			
Poor knowledge	1		
Fair knowledge	0.88	(0.50;1.54)	0.644
Good knowledge	1.31	(0.37;4.66)	0.673

^*^Significance at P-value < 0.05

The final adjusted model revealed that the odds of having injuries among children of currently employed mothers are almost three times higher than those of currently unemployed mothers while controlling for the mothers' practices towards child injury prevention (Table 6).

**Table 6:** The unadjusted and adjusted ORs for the dependent, independent, and other covariate variables

Variable	Unadjusted OR (95% CI)	*p*-Value	Adjusted OR (95% CI)	*p*-Value
*Current working mothers (Yes/No)*				
No	1		1	
Yes	2.61 (1.51;4.52)	0.001*	2.95 (1.60;5.47)	0.001*
*Mothers' practice*				
Poor practice	1		1	
Fair practice	1.19 (0.68;2.11)	0.676	0.79 (0.41;1.49)	0.413
Good practice	0.23 (0.07;1.49)	0.149	0.22 (0.05;1.00)	0.05*

*Significant at *p*-value < 0.05

## Discussion

To our knowledge, this is the first study conducted in Lebanon that assesses the mothers’ KAP toward child injuries. The study illustrated the type, characteristics, and patterns of child injuries and further examined their association with socioeconomic factors that increase the risk of injury among children.

This study reported the high prevalence of child injuries in Lebanon among young children (≤ 10 years old). While the disclosed results are consistent with regional rates reported in previous studies conducted in Saudi Arabia and India (15 to 30%) (Bashour and Kharouf 2018; Sousa et al. 2010; Gad et al. 2011; Gonçalves et al. 2019; Mathur et al. 2018), it is relatively higher than rates reported in developed countries (9 to 10%) (Otters et al. 2005; Kohen et al. 2000). Findings from this study revealed that the majority of injuries were sustained by males and children aged between 5 and 10 years, compared to those less than 5 years of age. Such outcomes align with previous observations reported in local studies (Al-Hajj et al. 2020a; Nuwahid et al. 2002), regional studies (Halawa et al. 2015), and international literature (Mathur et al. 2018; Borse et al. 2009). A plausible explanation can be attributed to the increased participation in outdoor activities, age-dependent curiosity, and a noticeably more risky behavior displayed among males and children aged 5–10 years (Mahalakshmy et al. 2011).

As for injury mechanisms, falls were responsible for the majority of childhood injuries, succeeded by burns. A consistent pattern was reported in previous studies conducted in Oman (Mehmood et al. 2016), Saudi Arabia (Al-Zahrany et al. 2018), and the United Arab Emirates (Grivna et al. 2015), revealing that falls represent the leading cause of childhood injuries, imposing a substantial toll on the children population, particularly among young children aged 0–5 years old. Burns were reported as the second leading cause of childhood injuries (Mehmood et al. 2018; Begum 2019; Mack and DeSafey 2010), similar to existing studies. Specifically, our results showed that burns were more pronounced among females aged 0–5 years. This disparity between males and females stems from the more frequent presence of females in the kitchen due to their nature and interest, in addition to the cultural norms and engagement in food cooking and serving (Lasi et al. 2010). Sports-related injuries are commonly reported in children aged 5–10 years, specifically in males aged 7 years or older. Studies from Canada, the United States, and Australia yielded similar patterns of sports injuries among school-aged children between 6 and 10 years (Kohen et al. 2000; Patel et al. 2017; Schneuer et al. 2018). The prevalence of such injuries calls for the establishment of safe indoor and outdoor play areas that satisfy international safety standards (Mutto et al. 2011).

The severity of the sustained injury was assessed by the medical attention it required. This present study indicated that 1 in 5 children required Emergency Department visits or hospitalization post-injury, namely due to falls and cuts. Hospitalized children were mostly males older than 5 years. This is consistent with a study conducted in Australia that revealed almost a double injury-related hospitalization rate among males compared to females (2362 compared to 1327 per 100,000), mainly due to falls (Leeds et al. 2015). A similar study conducted in low- and middle-income countries showed that fall injuries were the leading cause of childhood hospitalization (Hyder et al. 2009).

When examining mothers’ KAP scores related to child injury prevention, the reported scores revealed that mothers of children below 10 years have a fair knowledge of child safety, fair attitude and awareness, and poor practice of child injury preventive measures. Despite the uniqueness of our cohort characterized by highly educated mothers working in the health-related sector, and residing in an urban setting, this study clearly underscores the adverse lack of knowledge of child safety and injury prevention in this population. This finding resonates with previous studies conducted in Egypt, Iraq, and Ghana, where mothers demonstrated poor to fair KAP scores, reflecting low to moderate levels of knowledge of child injury prevention (Eldosoky 2012; Aldoori and Abed 2017; Sackitey 2018). Our findings presented robust evidence and supported the need for parental/caregivers’ child safety educational programs and awareness campaigns aiming at reducing and preventing child injuries.

Even though most injuries are predictable and preventable, an overwhelming number of mothers believed that fate played a decisive role in their children’s injury occurrence (Forward and Loubani 2018). On the other hand, many mothers blame themselves for their children’s injuries. Besides feeling responsible for safeguarding their child’s health and safety, cultural perception and social norms further induce this sense of accountability and feeling of self-guilt. These mothers perceptions revealed the willingness to change behavior at the societal level, improve self-efficacy towards changing lifestyle, seek knowledge about child injury prevention, and ask for support to acquire the needed knowledge and practices.

In terms of good practices towards child injury prevention, a small number of mothers demonstrated good practices of child home safety. While good road safety practices were reported by the majority of mothers, only 37% of the mothers used a child restraint system. This is consistent with the parental behavior in Saudi Arabia and in India, where studies showed the absence of a child restraint system and lack of compliance with road safety measures, such as using seatbelts (Verma 2016; AlSallum et al. 2019). Furthermore, despite being illegal, many of the surveyed mothers reported using their cell phones while driving with their children on board.

Lastly, this study indicates the association between child injuries and the mothers’ working status. Previous studies have reported that children of working mothers are at higher risk of sustaining injuries compared to non-working mothers, especially at-home injuries (Chaveepojnkamjorn et al. 2002; Faruque and Khan 2016). This significant association serves to highlight the fact that substitute caregivers may have limited childcare experience compared to mothers, particularly as working mothers have a better understanding of child safety and injury prevention concepts. This association further underscores the effect of substitute caregivers supervision practices on the child in the absence of a mother, especially that in many cases these caregivers prioritize house chores over child direct supervision and care (Sackitey 2018; Akturk and Erci 2016). Child supervision is a complex task, and proximity and attention are two dimensions likely related to injury risk and possible prevention. Inadequate active child supervision could be a reason for the increased likelihood of injury occurrence. This study lacks any details on the profile of caregivers in charge of supervising the child while mothers are at work. Generally, in the Lebanese culture, substitute caregivers are usually grandparents or domestic workers, whereas the latter are also responsible for other chores (i.e., cleaning and cooking). Nonetheless, other studies demonstrated the lack of any significant association between mothers' occupation and child injury occurrences, depending upon the context and settings (Eldosoky 2012; Karatepe and Akış 2013).

This study sheds light on an important social debate in Lebanese society about family members' role and responsibility towards child injury prevention and how mothers' knowledge attitudes and practices influence the safety of their children within and outside of households. Considering the common mechanism of injuries among children in this study, it is evident that mothers play a significant role, nonetheless, other caregivers including fathers, grandparents, and house helpers must be integrated into injury prevention strategies and education targeting behavioral changes, promoting injury-safe environments at houses, schools, in playgrounds, and during transportation.

A series of key takeaway messages about injury prevention and child supervision from this study: (1) The mothers’ practice of preventive measures of child injury was significantly associated with a lower occurrence of injuries; this should pave the way for further longitudinal studies that examine this phenomena with further scrutiny (2) Higher education status of women in Lebanon did not translate into good injury prevention knowledge or practices, thus requires targeted campaigns that focus on both the mothers and the substitute caregivers; (3) Children of working mothers were more likely to get injured owing to the inadequate supervision at home; highlighting the need for bringing other everyday caregivers including elder siblings, nannies or elderly grandparents, as well as the need for child injury prevention campaigns that must be framed in an inclusive language and signifies the role of caregivers and emphasize strategies directed at different individuals responsible for child caring (4) With the lack of child restraint system enforcement in Lebanon, mothers showed little compliance with the existing child safety laws. Nonetheless, child restraint should be viewed and practiced as a collective responsibility of both parents as well as those who bear the responsibility of transporting children in cars, taxis, and school buses.

When designing injury prevention programs in Lebanon, a major shift in parental belief that espouses preventability and predictability of injuries is needed to impact a behavior change. Both parents and caregivers must be encouraged to adopt safety measures to reduce child injuries. Child supervision methods, caregivers’ interactions, and practices should not be the sole responsibility of mothers, as the study highlighted a significant prevalence of mothers’ self-blame after an injury. Other models of supervision for preschool children such as creches and after-school adult supervision of older children must be considered for working mothers. These findings warrant the urgent need to educate parents about child restraint systems, enforce driving laws and child restraint regulations as well as restrict the use of any handheld devices while driving. Evidence from this study should be translated into tailored parental child safety interventions and preventive strategies that are effective in mitigating childhood injuries. Communication with health professionals, policymakers, and the Ministry of Labor should be initiated to set priorities and introduce effective workplace family-friendly policies (i.e., long maternity paid leaves, remote work, and on-site childcare facilities). These policies, proven effective in North America, allow mothers to have flexible work schedules and facilitate their direct and close supervision of their young children (Ruhm 2011).

The present study has several strengths. The study sample size is sufficient to reveal a significant association between variables. Accounting for the clustering effect i.e., the household where siblings who shared common factors were treated as independent individuals, is considered another strength. The adopted tool was comprehensive, covering common household injuries while capturing details on mothers’ socioeconomic status, child’s injury history, and mothers’ KAP towards child injury prevention. Moreover, the tool was contextualized and translated into the local language for more accurate responses.

This study has some limitations. First, despite the comprehensive approach to assessing mothers’ KAP about childhood injuries, several essential pieces of information were lacking from the KAP survey including injury location, safe home environment, and the profile of caregivers in charge of childcare while mothers were at work. Future studies warrant the inclusion of such information with their significant implications on the development of child safety interventions. Second, minor injuries that were managed at home without the need for an emergency department visit or hospital admission might be missing from this study due to a possible recall bias among participating mothers. Third, participating mothers may have denied the occurrence of child injury due to social desirability and to avoid blame on their part, which can lead to information bias and under-reporting of the actual burden. Fourth, the low response rate among less-educated mothers might introduce selection bias, thus impacting the external validity of the study and potentially inflating the prevalence of childhood injuries among highly educated and working mothers. This variation in the significant association between the mother’s working status and the risk of child injury may be attributed to the small sample size of the participating mothers which was insufficiently powerful to detect this strong association while controlling for the effect of possible confounders. The sample size is another element that makes it hard to generalize the findings of this study. Finally, this present study was conducted only in Beirut. While the results may be representative of the urban Lebanese population, there might be considerable variation in the mother’s educational level, socioeconomic status, and household environments in other settings including other parts of Lebanon.

## Conclusion

Childhood injury remains a major public health problem in the Eastern Mediterranean region, including Lebanon. It is responsible for a substantially high prevalence of child morbidity and mortality. Mothers were shown to have limited knowledge, attitude, and poor practices of child injury prevention. Concerted efforts are required to design and develop evidence-based educational programs to educate mothers and impart the needed knowledge and necessary skills to help prevent and control childhood injuries. Further research warrants the assessment of the childhood injury burden, its determinants and risk factors, and the extent of this major health problem at a national level.

## Data Availability

The datasets generated during and/or analyzed during the current study are available from the corresponding author on reasonable request.

## Appendix

See Table

**Table 7:** This table specifies test that was used and why, the variables included in the analyses, and the statistical tests adopted

Test	Reason for use	Independent Variables	Dependent variables	*p*-Value
Chi-square test	To measure the association between the occurrence of child injuries and the mothers’ working status	Mother’s working status	Occurrence of child injuries	< 0.01
Simple logistic regression	to determine the unadjusted odds ratio (OR) and 95% confidence interval (CI), and to obtain the p-values, accounting for the clustering effect (household)	Mother’s working status Educational level Family income	Occurrence of child injuries	< 0.01 < 0.01 < 0.01
Multiple logistic regression	to obtain the adjusted ORs	Mother’s working status	Occurrence of child injuries	< 0.01

^7^.

## Abbreviations

KAP: Knowledge, attitude, and practices
UNRWA: United Nations Relief and Works Agency for Palestine Refugees
DALYs: Disability-adjusted life years
IRB: Institutional Review Board
OR: Odds ratio
CI: Confidence interval
